# Complex PTSD Pathway for Kingfisher Mother and Baby Unit

**DOI:** 10.1192/bjo.2024.384

**Published:** 2024-08-01

**Authors:** Jemima Jackson

**Affiliations:** Norfolk and Suffolk NHS Foundation Trust, Norwich, United Kingdom

## Abstract

**Aims:**

When Kingfisher Mother and Baby Unit (MBU) opened in 2019 personality disorder and severe self-harming behaviours were exclusion criteria for admission. Complex Post Traumatic Stress Disorder (C-PTSD) with its emotional dysregulation, interpersonal difficulties and common presence of self-harm was similarly categorised.

Currently, C-PTSD presentations are frequently admitted to the MBU, making up around 45.9% of admissions. There is increasing understanding of the importance of effective and trauma informed treatment in admission outcomes, particularly in reducing trans-generational trauma. This requires appropriate skills and training in staff.

**Methods:**

Service users were identified retrospectively over a 24-month period and categorised into C-PTSD traits (trait) and non-C-PTSD traits (non-trait). Comparisons of routine outcome measures (ROMs) identified higher distress in the C-PTSD group and reduced satisfaction. Staff survey highlighted areas of anxiety and low confidence in working with service users with C-PTSD traits.

Actions were divided into three streams – Admission, Transitions and Communication. Staff training needs were identified and training given. Admission processes were reviewed with a focus group including past service users and changes based on DBT principles were implemented. A leaflet was developed to aid communication with service users considering MBU admission via Outreach and Community Perinatal teams.

**Results:**

Surveys were the primary source of data before and after changes. As of September 2023 the majority of training had been rolled out but numbers completing the training survey were too small to draw conclusions. Anecdotal feedback was predominantly positive and the survey will be repeated at the same time as other data in March 2024.

Ward process changes started in late August 2023 and routine outcome measure data will be compared at 6 months (March 2024). Again anecdotal feedback is positive.

The leaflet was rolled out for use by community teams and service users in November 2023 and feedback via survey will be collected in March 2024.

**Conclusion:**

Evaluation of routine outcome measures showed poorer outcomes and experiences for patients with traits of Complex-PTSD. Staff survey highlighted lack of confidence in managing the same. Consultation with a range of staff and past service users led to changes in admission practices, communication prior to admission via a leaflet, and staff training. Anecdotal feedback since implementation has been positive but we hope to see this confirmed in the Routine Outcome Measures and surveys 6 months after the changes were implemented.

